# Height and Weight Bias: The Influence of Time

**DOI:** 10.1371/journal.pone.0054386

**Published:** 2013-01-23

**Authors:** Frances Shiely, Kevin Hayes, Ivan J. Perry, C. Cecily Kelleher

**Affiliations:** 1 Department of Epidemiology and Public Health, University College Cork, Cork City, Ireland; 2 Department of Mathematics and Statistics, University of Limerick, Limerick, County Limerick, Ireland; 3 School of Public Health, Physiotherapy and Population Sciences, University College Dublin, Dublin City, Ireland; Foundation for Liver Research, United Kingdom

## Abstract

**Background:**

We have previously identified in a study of both self-reported body mass index (BMI) and clinically measured BMI that the sensitivity score in the obese category has declined over a 10-year period. It is known that self-reported weight is significantly lower that measured weight and that self-reported height is significantly higher than measured height. The purpose of this study is to establish if self-reported height bias or weight bias, or both, is responsible for the declining sensitivity in the obese category between self-reported and clinically measured BMI.

**Methods:**

We report on self-reported and clinically measured height and weight from three waves of the Surveys of Lifestyle Attitudes and Nutrition (SLÁN) involving a nationally representative sample of Irish adults. Data were available from 66 men and 142 women in 1998, 147 men and 184 women in 2002 and 909 men and 1128 women in 2007. Respondents were classified into BMI categories normal (<25 kg/m^2^), overweight (25–<30 kg/m^2^) and obese (≥30 kg/m^2^).

**Results:**

Self-reported height bias has remained stable over time regardless of gender, age or clinical BMI category. Self-reported weight bias increases over time for both genders and in all age groups. The increased weight bias is most notable in the obese category.

**Conclusions:**

BMI underestimation is increasing across time. Knowledge that the widening gap between self-reported BMI and measured BMI is attributable to an increased weight bias brings us one step closer to accurately estimating true obesity levels in the population using self-reported data.

## Introduction

We have previously identified in a study of both self-report body mass index (BMI) and clinically measured BMI that the underreporting of BMI has significantly increased across time. We also reported a decrease in the sensitivity score in the overweight and obese category over time, more significantly in the obese category (80%→64%→53%) in this 10-year period from 1998 to 2007 [1]. It is known that self-reported weight is significantly lower than measured weight for both men and women [2]–[7] and that self-reported height is significantly higher than measured height in adults [2], [4], [5], [7], [8]. It is not known if this self-report bias is consistent across time or if a change in self-reporting height bias or a change in self-reporting weight bias, or both, is responsible for the declining sensitivity in the obese category.

A plethora of literature exists on the validity of self-reported weight and height with the general conclusion that overweight and obesity, as determined from body mass index (BMI) categories, are generally underestimated when calculated from self-reported data compared to measured data [3], [7]–[22]. These findings have been derived primarily from data collected in Western cultures, and one study in Australia. However, some Asian studies, specifically Korea and Japan, have also reported the same conclusion [22], [23], though the magnitude of the bias is small. Caution must be applied in the case of the Japanese study as it was confined to adults over 70 years only. The importance of obesity as a public health issue, with well documented links between excess weight and disease, has been well cited [5], [8], [21], [24].

Two recent studies have examined the bias in self-reported and measured BMI across time in the USA (three time points; 1976–1980, 1988–1994, 2003–2004), Canada (two time points; 1986–1992, 2005) and Australia (two time points; 1995, 2008) [21], [25]. Height and weight bias as well as BMI bias were all examined separately but exclusively from their BMI misclassification. Findings from all three countries included in the studies differed, leaving no definitive conclusion as to whether the bias is constant or changing systematically over time. The Canada-US study highlights the importance of establishing if the self-reporting bias, evident in most self-report population surveys, is constant, systematically changing or randomly changing over time. A third recently published study [26], also conducted on the US-National Health and Nutrition Examination Survey (NHANES) dataset, as was the US Canada study [21], but including a more recent time point (two time points; 1998–1994, 2005–2008), concluded that the bias in self-reported height and weight has declined, leading to more accurate BMI categorisations based on self-report. This however is at odds with the US-Canada study, conducted on the same dataset which reported a stabilising of self-reporting bias. In fact, closer examination of the paper shows that in the results section, the authors state that “as the mean discrepancies between self-reported and measured BMI scores show: for the 2005–2008 US resident population as a whole, self-reported BMI scores underestimate true BMI scores by a slightly larger amount than those associated with the 1998–1994 population”, thereby agreeing with the US-Canada study. Further examination of the methodology shows that there is an inherent confusion of terms. The authors assume that more accurate BMI estimates, i.e., improved sensitivity scores between the two time points, infers more accurate reporting of both height and weight. This may not be the case and is the focus of our study.

It is our belief that height bias is constant and that weight bias is increasing. We hypothesise that the decrease in the sensitivity of the obese category over time in our recent study [1], is not due to an increase in self-reported height bias but due to an increase in self-reported weight bias as a result of the normalisation of obesity. To our knowledge this study presents the first focus on height and weight self-reported bias across time, in the context of BMI classification.

## Methods

### Ethics Statement

SLÁN 1998 and SLÁN 2002 were approved by the Faculty of Public Health Medicine, Royal College of Physicians of Ireland and SLÁN 2007 was approved by the Research Ethics Committee of the Royal College of Surgeons of Ireland.

### Participants and Data

The Survey of Lifestyle Attitudes and Nutrition (SLÁN) was first conducted in 1998 [27] (n = 6539), and repeated in 2002 [28] (n = 5992) and 2007 [29] (n = 10364). The methods have been described previously [1]. Briefly, the 1998 and 2002 surveys consisted of a multi-staged random sample using district electoral divisions (DEDs) across the 26 counties of the Republic of Ireland as the primary sampling units. A self-administered postal questionnaire was distributed to adults aged 18 years and over, and response rates of 62% and 53% were recorded.

The data used in this analysis were obtained from physical examination subsamples from three national health and lifestyle surveys in Ireland. In SLÁN 1998 and SLÁN 2002, the self-reported and measured height and weight data were obtained from an out of sample, 10% equivalent of the main postal survey, (SLÁN 1998 n = 586; SLÁN 2002 n = 411). This approach was taken for a variety of logistical reasons, including the fact that a) interviewers were not visiting the respondents at home and b) a random subgroup examination at home would be difficult to achieve on a cross-country basis. Research nurses were in attendance for all screening clinics and the examinations were undertaken using the standard European Health Risk Monitoring protocol. In 1998, 16 additional district electoral divisions, 8 on the Western seaboard and 8 on the Eastern seaboard of Ireland were randomly selected for the examination study. The required number of participants was set for each DED location based on its population size, and a target of 1359 participants in total was set. Potential respondents in these selected DEDs received a letter of invitation to attend a designated clinic for examination and to bring with them the completed self-administered questionnaire. In the event 586 were seen, 43.1% of the original targeted number. However, if we assume 10% non availability, as was a minimum established through return to sender in the main surveys, the minimum corrected response rate is 47.9%. If we also assume 40.78% non availability, as was the case in 2002, the response rate would be 72.79%.

In 2002 an additional separate sample was randomly selected for the examination component and this was undertaken in 13 district electoral divisions, 7 urban and 6 rural. The reasons for non-response were recorded, in that if the person receiving the letter did not reply by either refusing to participate or agreeing to an appointment, a visit was made to the household and the outcome of that visit was coded. Of 1118 dispatched letters, 411 examinations were achieved. Just 258 (23.01%) refused outright the invitation giving an uncorrected response rate of 30%, but when combined non-contactables (for a variety of reasons including moved away, deceased, unavailable for appointment) are removed, the response rate was 50.3%. A further comparison of these out of sample respondents with the main surveys indicated no significant difference in key variables such as age, level of secondary educational attainment and smoking status. In all four datasets males were under-represented, significantly so in 1998.

SLÁN 2007 consisted of a probabilistic sample in three stages – geographic area, household and ‘next birthday’ participant selection within households. The sample frame was the Geodirectory, a listing of all residential address in Ireland compiled by the postal service. Face-to-face interviews were conducted with adults aged 18 years and over interviewed at home addresses (response rate of 62%). All participants were asked to self-report their weight without clothes and their height without shoes. Examination data were obtained on an approximate 20% subsample, n = 2174. Respondents provided self-reported data at interview before they were asked to agree to have their height and weight measured. Weight and height were measured in light clothing without shoes. Weight was measured to the nearest 0.1 kg using electronic platform scales. Height was measured to the nearest 0.1 cm using height measurement rods. Data were missing, we assume at random, for some measured weight, measured height, self-reported weight and self-reported height variables in each of the SLÁN surveys leading to 208 (M 66; F 142), 331 (M 147 F 184) and 2037 (M 909 F 1128) complete cases for comparison respectively in 1998, 2002 and 2007.

### Statistical Methods

**Figure 1:**
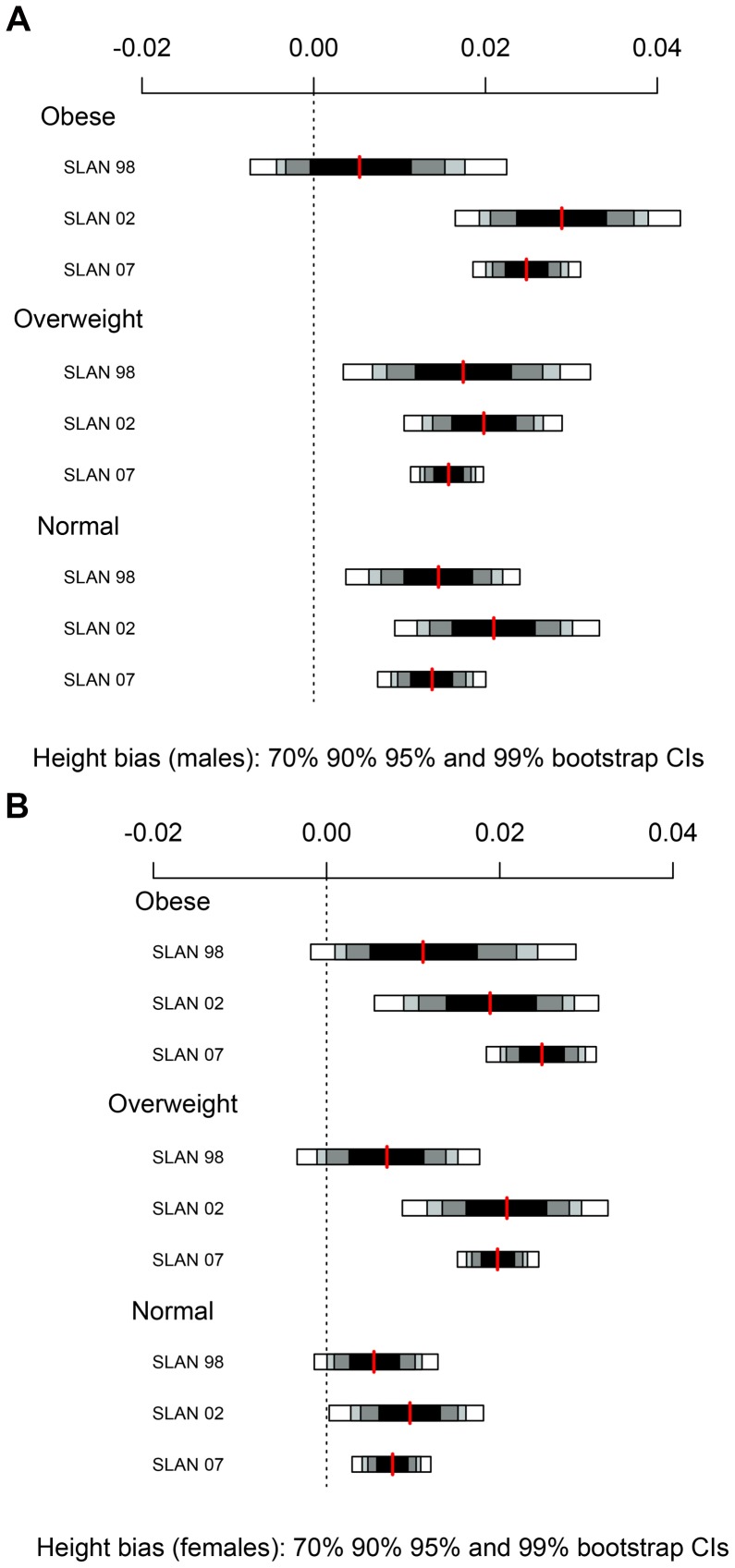
Self-reported height bias by clinically measured BMI category with 70%, 90%, 95% and 99% bootstrap confidence intervals.

**Figure 2:**
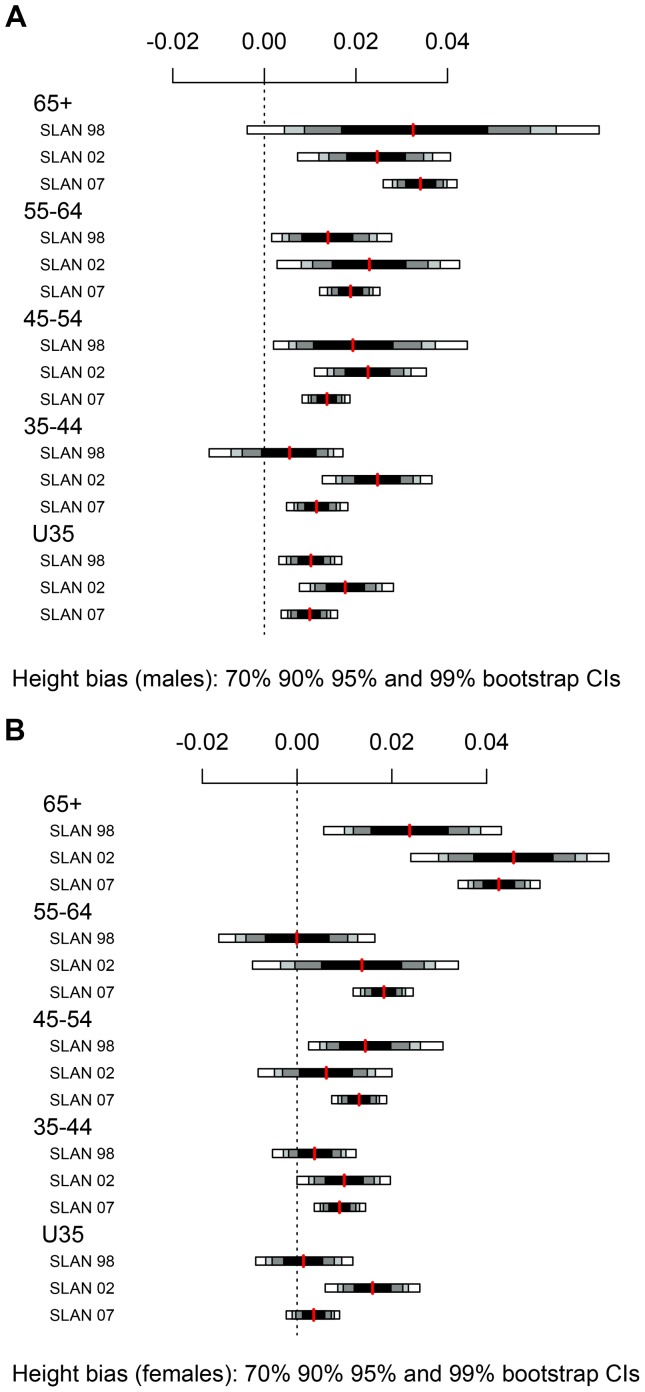
Self-reported height bias by age category in SLÁN 1998, SLÁN 2002 and SLÁN 2007.

**Figure 3:**
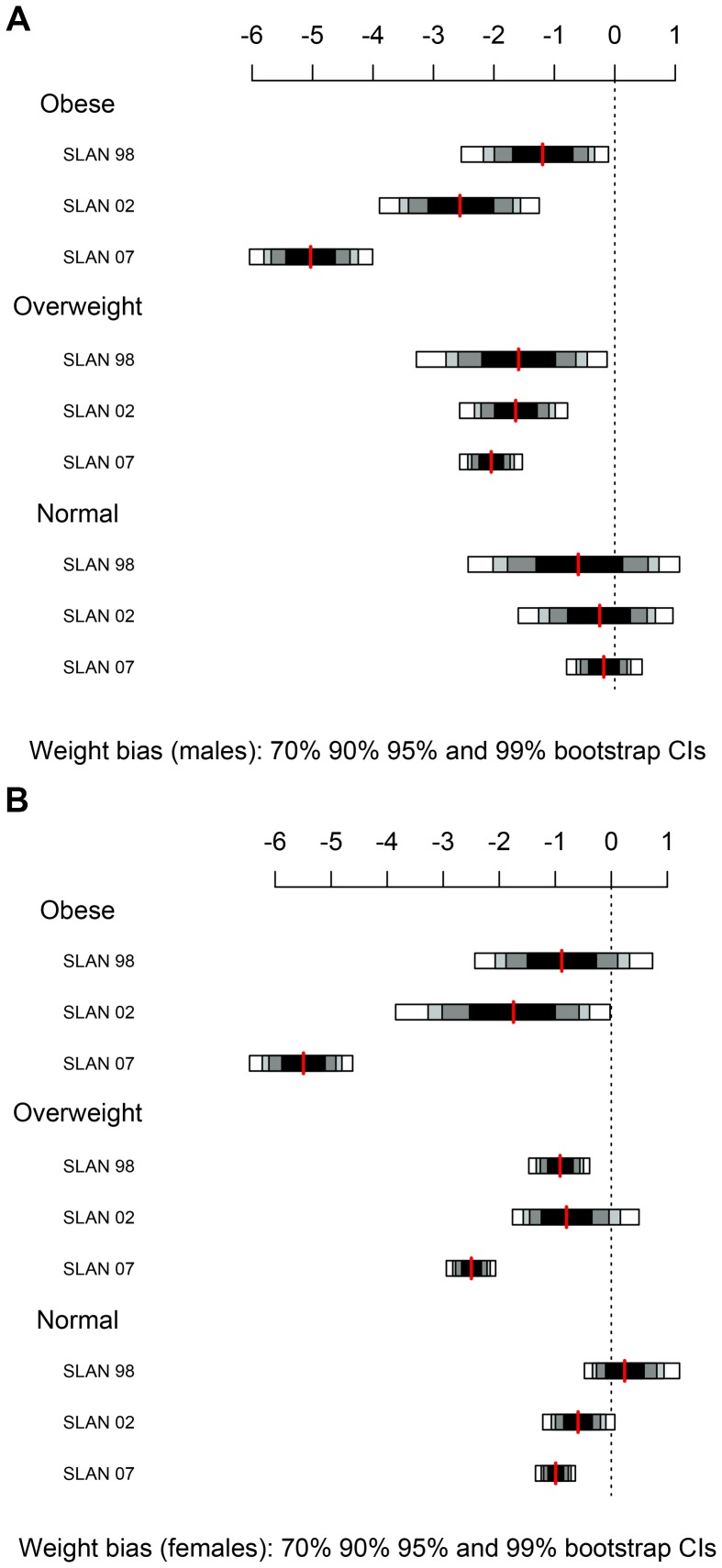
Self-reported weight bias by clinically measured BMI category with 70%, 90%, 95% and 99% bootstrap confidence intervals.

**Figure 4:**
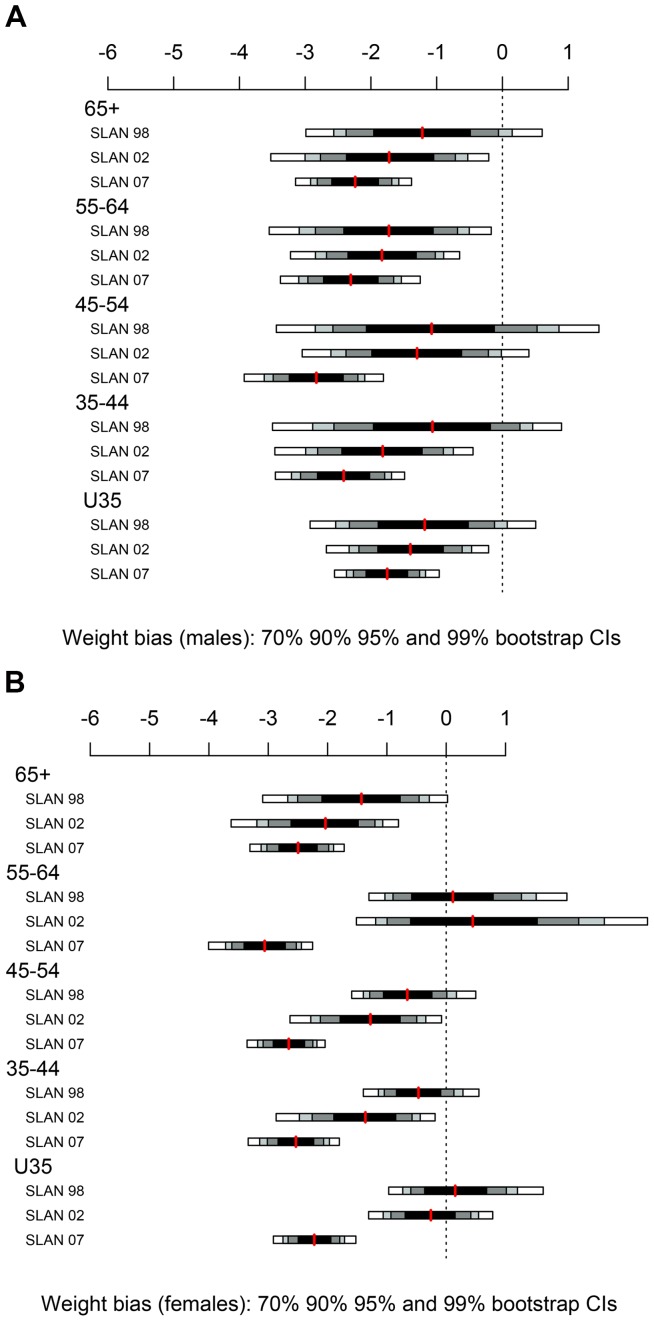
Self-reported weight bias by age category in SLÁN 1998, SLÁN 2002 and SLÁN 2007.

Self-report height (weight) bias was calculated by subtracting the clinically measured height (weight) from the self-reported value height (weight). Graphical views (histograms, boxplots, normal quantile-quantile plots) of the height and weight reporting biases for both males and females, across age subgroups and true BMI subgroup, displayed much evidence of non-Gaussian, asymmetrical long-tailed distributions. The Shapiro test for Gaussian distributed data was applied to the reporting biases and was rejected (p<0.05) in over 60% of these subgroups. To account for this, bootstrap 70%, 90%, 95% and 99% confidence intervals for the mean height and weight biases were calculated for each subgroup and are displayed in figures 1, 2, 3 and 4 and tables 1 and 2. All statistical analysis was performed using R 2.8.1 [30].

**Table 1:** Mean height bias and confidence intervals, classical (top) and bootstrap (second row), between self-reported and measured heights by age for male subjects and female subjects in SLÁN 1998, SLÁN 2002 and SLÁN 2007.

Male	SLÁN 1998					SLÁN 2002					SLÁN 2007				
	Height bias	N	SD	P	95% CI	Height bias	N	SD	P	95% CI	Height bias	N	SD	P	95% CI
**<35**	0.01	18	0.012	0.002	0.004, 0.016	0.018	26	0.021	0	0.009, 0.026	0.01	229	0.037	0	0.005, 0.015
					0.005, 0.015					0.01, 0.026					0.005, 0.014
**35–44**	0.005	18	0.025	0.364	−0.007, 0.018	0.025	35	0.028	0	0.015, 0.034	0.011	165	0.033	0	0.006, 0.016
					−0.007, 0.015					0.016, 0.034					0.006, 0.016
**45–54**	0.019	12	o.o3	0.048	0, 0.038	0.023	32	0.027	0	0.013, 0.032	0.014	177	0.027	0	0.01, 0.018
					0.005, 0.037					0.014, 0.032					0.01, 0.018
**55–64**	0.014	10	0.018	0.035	0.001, 0.027	0.023	31	0.044	0.007	0.007, 0.039	0.019	145	0.03	0	0.014, 0.024
					0.004, 0.025					0.008, 0.038					0.014, 0.024
**65+**	0.032	8	0.046	0.085	−0,006, 0.071	0.025	23	0.031	0.001	0.011, 0.038	0.034	193	0.043	0	0.028, 0.04
					0.004, 0.063					0.012, 0.037					0.028, 0.04
**Female**															
	**Height bias**	**N**	**SD**	**P**	**95% CI**	**Height bias**	**N**	**SD**	**P**	**95% CI**	**Height bias**	**N**	**SD**	**P**	**95% CI**
**<35**	0.001	26	0.02	0.74	−0.007, 0.01	0.016	46	0.026	0	0.008, 0.024	0.004	262	0.036	0.12	−0.001, 0.008
					−0.07, 0.009					0.008, 0.023					−0.001, 0.008
**35–44**	0.004	52	0.025	0.286	−0.003, 0.011	0.01	51	0.027	0.012	0.002, 0.018	0.009	229	0.032	0	0.005, 0.013
					−0.003, 0.01					0.003, 0.017					0.005, 0.013
**45–54**	0.014	35	0.032	0.013	0.03, 0.026	0.007	42	0.034	0.174	−0.003, 0.018	0.013	256	0.036	0	0.009, 0.018
					0.005, 0.026					0.003, 0.017					0.008, 0.018
**55–64**	0	19	0.029	0.994	−0.014, 0.014	0.014	22	0.039	0.117	−0.004, 0.031	0.018	191	0.033	0	0.014, 0.023
					−0.013, 0.012					−0.004, 0.029					0.014, 0.023
**65+**	0.024	8	0.023	0.022	0.005, 0.043	0.046	23	0.039	0	0.029, 0.062	0.043	190	0.045	0	0.036, 0.049
					0.009, 0.039					0.03, 0.06					0.036, 0.049

**Table 2:** Mean weight bias and confidence intervals, classical (top) and bootstrap (second row), between self-reported and measured weights by age for male subjects and female subjects in SLÁN 1998, SLÁN 2002 and SLÁN 2007.

Male	SLÁN 1998					SLÁN 2002					SLÁN 2007				
	Weight bias	N	SD	P	95% CI	Weight bias	N	SD	P	95% CI	Weight bias	N	SD	P	95% CI
**<35**	−1.182	18	2.907	0.103	−2.627, 0.264	−1.14	26	2.479	0.008	−2.401, −0.398	−1.752	229	4.705	0	−2.365, −1.139
					−2.479, 0.064					−2.348, −0.437					−2.396, −1.151
**35–44**	−1.064	18	3.825	0.254	−2.966, 0.838	−1.821	35	3.475	0.004	−3.014, 0.627	−2.415	165	5.068	0	−3.194, −1.635
					−2.949, 0.528					−2.99, −0.744					−3.226, −1.65
**45–54**	−1.077	12	3.427	0.3	−3.524, 1.101	−1.298	32	3.752	0.059	−2.651, 0.054	−2.83	177	5.171	0	−3.597, −2.063
					−2.786, 0.866					−2.599, −0.067					−3.642, −2.073
**55–64**	−1.726	10	2.19	0.034	−3.293, −0.159	−1.833	31	2.835	0.001	−2.872, −0.793	−2.306	145	4.841	0	−3.101, −1.512
					−3.066, −0.511					−2.868, −0.884					−3.113, −1.498
**65+**	−1.217	8	2.099	0.145	−2.973, 0.538	−1.723	23	3.144	0.015	−3.082, −0.363	−2.239	193	4.771	0	−2.916, −1.562
					−2.586, 0.251					−3.12, −0.54					−2.912, −1.58
**Female**															
	**Weight bias**	**N**	**SD**	**P**	**95% CI**	**Weight bias**	**N**	**SD**	**P**	**95% CI**	**Weight bias**	**N**	**SD**	**P**	**95% CI**
**<35**	0.152	26	2.559	0.765	−0.882, 1.185	−0.259	46	2.788	0.533	−1.087, 0.569	−2.224	262	4.29	0	−2.746, −1.702
					−0.749, 1.2					−1.038, 0.539					−2.748, −1.721
**35–44**	−0.467	52	2.632	0.208	−1.203, 0.268	−1.362	51	3.737	0.012	−2.413, −0.311	−2.533	229	4.419	0	−3.109, −1.958
					−1.111, 0.256					−2.454, −0.412					−3.118, −1.975
**45–54**	−0.656	35	2.353	0.108	−1.464, 0.152	−1.305	42	3.088	0.009	−2.268, −0.343	−2.654	256	4.145	0	−3.164, −2.144
					−1.41, 0.134					−2.293, −0.395					−3.157, −2.155
**55–64**	0.133	19	2.967	0.87	−1.317, 1.543	0.448	22	4.9	0.673	−1.725, 2.62	−3.06	191	4.582	0	−3.714, −2.406
					−1.049, 1.567					−1.192, 2.794					−3.717, −2.47
**65+**	−1.429	8	1.874	0.068	−2.996, 0.138	−2.035	23	2.681	0.001	−3.195, −0.876	−2.496	190	4.313	0	−3.114, −1.879
					−2.728, −0.295					−3.201, −1.078					−3.102, −1.904

## Results

### Height

Self-reported height bias was calculated and male and female subjects were categorised according to their clinically measured BMI category (Figures 1a and 1b). The self-reported height bias is positive at all times indicating that height is over reported. Inflating the numerator in this manner leads to an underestimation of BMI. The scale on the plot in Figures 1a and 1b shows that the magnitude of the self-reported height bias is quite small for both males and females, and is systematic across all BMI categories and across time, evidenced by the overlapping confidence intervals.

It is well known that older adults, particularly females, overestimate their height [8], [10], [11], [13]. We investigated the influence of age on self-reporting bias. Male and female subjects were categorised by the age categories, <35 years, 35–44, 45–54, 55–64 and 65+ and self-reported height and weight bias was plotted (Figures 2a and 2b). In all age groups and for both genders, the self-reported height bias was positive. Female height reporting bias was constant across time, evidenced by the overlapping confidence intervals, but not constant across age groups. Older women overestimate their height more than younger women. This can be seen from the confidence intervals in Table 1 and is also demonstrated by the plots in Figure 2b. The extremely small numbers in the older age categories account for the wide confidence bands. Males overestimated their height in all age groups and this overestimation was also stable across time (Figure 2a). Contrary to females, the reporting bias was constant across all age groups (Table 1 and Figure 2b).

### Weight

The 95% confidence intervals for the self-reported weight bias for each clinically measured BMI category are shown in Figures 3a and 3b for males and females respectively. The red line represents the mean weight for each SLÁN survey in the normal, overweight and obese BMI categories. With the exception of normal weight females in SLÁN 1998, the self-reported weight bias is negative indicating that weight is underreported. This leads to an underestimation of BMI. Most notable in Figures 3a and 3b is that the self-report weight bias dramatically increased across time in the obese category, for both males and females. Note the separation of the confidence bands, particularly between SLÁN 2002 and SLÁN 2007. For females in the overweight category, this trend is also observed between the latter two time points. For males, the self-reported weight bias in the overweight and normal categories is stable across time. Self-report weight bias in the normal category was stable across time for both genders.

The 95% confidence intervals for the self-reported weight bias for each age group taken individually are shown in Table 2 and Figures 4a and 4b. For males and females in SLÁN 1998 all the confidence intervals (except one) include zero, indicating no systematic reporting error. The confidence intervals for SLÁN 2002 and SLÁN 2007 are entirely negative suggesting that weight is underreported in each age group. As shown in Figure 4a and 4b, for both sexes, self-report weight bias increases across time but is consistent across age group.

## Discussion

### Principal Findings

As the use of self-reported data to classify obesity continues, the sources of the reporting errors remain unclear. These data from three nationally representative population surveys show that self-reported height bias is stable over time regardless of gender, age or clinically measured BMI category. Self-reported weight bias is continuing to increase regardless of gender or age or knowledge that weight would be measured after self-reporting. These data further show that when classified by clinical BMI measurement, across time, the increase in self-reported weight bias is evidenced only in the obese category for males and the obese and overweight categories for females.

### Comparison with Other Studies

A focused search of the BMI literature revealed only one study which focused on height and weight across time [25], but this study did not look at these biases in the context of BMI category. The search identified many research articles devoted to the validity of self-reported height and weight [3], [7], [8], [10]–[18], [22], [31], [32]. While not all focus on both height and weight, all express a similar opinion, i.e., an underestimated numerator and an overestimated denominator lead to a pattern of underestimation when self-reported height and weight are used to calculate BMI. The fact that older adults have been shown to systematically overestimate their height [7], [8], [10]–[14], and women tend to underreport their weight and men tend to overreport their weight [6], [11], [15], [17], [18], may have led researchers to accept that self-report height and weight bias both contribute equally to an underestimation of BMI. Two recent articles focusing on ethnic differences in self-reported and measured obesity add weight to this conclusion [22], [31]. The most recent publication on temporal trends in BMI bias [26] also comes to the same erroneous conclusion. Many studies have focused on trying to correct for this underestimation to provide a more accurate estimation of BMI, and particularly obesity prevalence, in populations [8], [25], [33], [34]. But the generalisation of these equations depends on the stability of the self-reporting bias over time and populations. Connor Gorber and Tremblay [21] recently suggested that if we can establish if the bias is constant or changing systematically over time, then self-reported estimates may still be valuable for monitoring trends and could be statistically adjusted to increase their accuracy. Our conclusions that self-report height bias remains stable over a 10 year period, and that self-report weight bias is increasing, but for the main in the obese category, will now allow researchers to apply imputation methods more accurately.

Our recent finding, using three population surveys from Ireland with both clinically measured and self-reported BMI [1], a decline in the sensitivity of the obese category across time (79.5%→64%→53.4%), led us to question if in fact both an overestimation of height and an underestimation in weight contribute equally to this decline in sensitivity over time. Our present study using all three BMI classifications and three cross sectional studies at three time points shows that the declining sensitivity in the obese category is caused by an increasing underestimation of weight, while the effect of height overestimation remains stable over time. On one hand, such inaccuracies could be understood as the consequence of a lack of information regarding one’s own height and weight. It is also possible that this group are in denial of their unhealthy weight, or don’t want to be labelled as obese. On the other hand, a more plausible explanation, given the rising clinically measured overweight and obesity levels in Ireland between SLÁN 1998 and SLÁN 2007 (60% to 64%) [1] is that increases in the adiposity levels of the general population may have normalised obesity. Recent literature suggests that there is a shift in the social norm of what is regarded as overweight or obese [9], [35]. Thus the height and weight errors evident here may be as a result of a cognitive distortion affecting the individuals’ perception of their own body shape [32]. This would explain why older individuals show a greater unawareness of their actual height and weight compared to other age groups. Survey context should also be given consideration. Just as prior knowledge of height and weight measurement leads to more accurate self-report estimates [36], giving information on the importance of accurate estimates to participants before they self-report their height and weight, may lead to more accurate responses.

A recent 2011 study based their study objectives on the assumption that social norms regarding what constitutes an ideal body weight also affect individuals’ self-reporting decisions when answering anthropometric questions on health questionnaires [32]. They report that, the greater the average “ideal” weight shared by the reference group, the lower the weight bias or the less inclined sample individuals are to under-report their weight. An important finding to support this theory in the current study is that while self-reported weight bias is evident in all three BMI categories, it is most notably on the increase in the obese category for both males and females (Figures 3a and 3b).

As in existing literature, we identified that female height reporting bias is not consistent across all age groups and that older women tend to overestimate their height more than younger women [8], [10]–[14]. An important additional finding is that this overestimation in height is consistent across time and also across clinically measured BMI category.

The underestimation of weight in the normal category is consistent across time. A recent British study identified a decline in the sensitivity of self-diagnosis of overweight [9]. A disadvantage of the study was the inability to distinguish between the overweight and obese BMI categories. An important finding in the current study, is the establishment that the underestimation of weight is stable across time for males in the overweight category but not so for females. While the underestimation of female weight in the overweight category was stable from 1998 to 2002, we observed an increase to 2007. As overweight levels, as well as obese levels, in the general population are on the increase, females that measure as overweight are finding it difficult to recognise they are overweight. It is important to monitor this trend in future population surveys.

The recent paper by Stommel and Osier [26] on temporal trends in BMI bias across time in the USA reported that the bias in self-reported height and weight has declined, leading to more accurate BMI categorisations based on self-report. This finding appears to be at odds with the findings of our study. However, the Stommel and Osier paper have examined BMI bias, not height bias or weight bias, and incorrectly assume improved accuracy in reporting of *both* height and weight. Nevertheless, if we focus on the improvement in BMI categorisations, as outlined by Stommel and Osier, what is calculated is the sensitivity of six BMI categories, underweight <18.5 kg m^−2^, normal weight 18.5<25 kg m^−2^, overweight 25<30 kg m^−2^, obese I 30<35 kg m^−2^, obese II 35<40 kg m^−2^ and obese III 40+ kg m^−2^. The sensitivity of the underweight, normal weight and overweight categories remains the same between the two time points. Improvements in the sensitivity of the obese categories are evident, and this improvement is greatest for those in obese category III, or >40 kg m^−2^ (54.4%→71.7%). We would argue that these patients are most likely engaged with the health service given their extremely high BMI, and their height and weight are therefore monitored regularly. Consequently, they will know their height and weight and this will result in a smaller self-report BMI bias. This is likely the source of the improvement in BMI categorisation, rather than a population wide improvement in self-reported height and weight. There are some limitations to our own findings, all three are relatively small samples and the two older surveys have modest response rates. Nonetheless we have established that in socio-demographic terms all three surveys are reasonably representative of the main datasets and all three surveys used a clustered random selection strategy countrywide, using standardised measurement protocols.

### Implications for Research and Practice

The findings in this study question other work that both self-reported height bias and self-reported weight bias contribute equally to BMI underestimation in large population surveys. Our results add significantly to the existing literature. Our findings also give considerable cause for concern – where accuracy is of prime importance, i.e. the obese category, self-reported weight biases are increasing. The magnitude of this increase should be monitored closely in the next round of the SLÁN surveys on a sample size similar to SLÁN 2007.

Sixty four percent of Irish adults are measured as overweight or obese [29], a significant health burden in the population. Though technically simple, obtaining accurate measurements of height and weight in large epidemiological studies is not practical. The International Obesity Task Force in its briefing on obesity in Europe makes the point that “few countries conduct systematic measured surveys to obtain reliable nationally representative data to assess the degree of overweight and obesity in their populations” [24]. There is then a need to correct self-reported surveys to obtain accurate measures of overweight and obesity [32]. The current study identifies that height overestimation is consistent across time for males and females and in all BMI categories. While self-reported height bias is greatest in older females (65+ years), this is consistent across time. This new finding allows for researchers to accurately adjust for this overestimation and hence lead to more accurate measurement of BMI in self-report surveys. No studies have reported on height bias in the context of BMI classification over time. However, the US-Canada study reported on height bias across time. Self-report height bias was 0.9 cm in 1976–1980 and just 0.8 cm from 2003–2004 in the US [21] and increased from just 0.4 cm to 0.8 cm in Canada between 1986 and 2005. Thus, height bias remains relatively stable across time in both of these countries. A study in Australia [25] reported that height bias decreased across time. However this result should be interpreted with caution as the mean height error was plotted against measured height, a methodology that induces a correlation between the plotted quantities [37]. Height bias therefore appears to be stable internationally. Further work is needed to extrapolate this finding to other countries. There is little doubt that access to reliable information is crucial to health policy makers dealing with the obesity epidemic in order that they can make appropriate public health policy responses.

While self-reported weight bias is consistent across age group and gender, identifying that this bias is greatest in the obese category and is increasing across time has important implications for future studies. Providing accurate estimates of actual height from self-reported height, and actual weight from self-reported weight should first be undertaken before attempting to estimate actual BMI from self-reported BMI. Accurate monitoring of height and weight are important in the diagnosis, prevention and reduction of overweight and obesity. The implications for future studies are clear, in large population surveys, project investigators should at a minimum obtain clinical measurements from a subsample of participants to monitor underestimation and overestimation trends.

### Strengths and Weaknesses of the Study

The results must be cautiously interpreted, in this analysis. We accept that numbers are small in both 1998 and 2002 sweeps for the out of sample component with a modest response rate, limiting both precision and generalisability, though non systematic factors such as logistics in attendance may be more at play here, rather than outright bias. The respondents are not self-selected, as stated in a recent publication [26]. The final numbers reported in the present submission were based on complete files, due to missing at random variables. However we are not reporting prevalence figures and are concerned in this analysis purely with a within-person validation by recorded measurements of self-reported information. The data do also have the advantage of being recorded to a strict protocol by trained nurses. There is no systematic reason to believe that the people who attended the clinic would be particularly biased compared to non-attenders in their self report of a given variable at one time point versus another, rather that there have been true secular changes in reported weight.

Data collection methods were not identical in the three surveys. SLÁN 1998 and SLÁN 2002 represent out of sample groups while the SLÁN 2007 groups are a subsample of the main survey. We have taken the confirmatory strategy of going back to the original datasets in order to compare the demographic profile of the examined respondents in both SLÁN 1998 and SLÁN 2002 with the main sample for age, sex, education, smoking status and both self-reported and examined body mass index. This shows that these out of sample respondents are reasonably comparable with the respondents in the main datasets. No significant age differences were seen between self-reported and examination data participants at either time-point, or no significant differences in smoking rates. In all four datasets more women than men participated, lowest in the 1998 examination data, with significantly fewer male participants in the examination survey in 1998. Self-reported BMI was significantly higher in examined participants at both time points but that may be explained by self-selection and does not alter the overall premise of the results.

While the response rates were 62%, 53% and 62% respectively for the three SLÁN surveys, information on the differences between responders and non-responders is not available for evaluating the potential bias due to non-response.

### Concluding Remarks

Obesity is a public health and policy problem because of its prevalence, costs and health effects. The findings in this study are significant for shaping future public policy around overweight and obesity awareness. We question other reported work that both an overestimation of self-reported height and an underestimation of self-reported weight contribute equally to an underestimation of BMI. Our findings suggest that self-report weight bias contributes in the main to the increasing underestimation of BMI. Coupled with rising obesity levels [1], the increasing self-reported weight bias in the obese category for both males and females is evidence of the normalisation of obesity in the Irish population. An increase in self-reported weight bias between 2002 and 2007 for females in the overweight category suggests that the normalisation of overweight is also evident and should be evaluated into the future.
